# ASIC1 Emerges as an ATC-Associated Candidate from an Integrative Dual miRNA-mRNA Analysis

**DOI:** 10.3390/biomedicines14102264

**Published:** 2026-10-07

**Authors:** Berrin Ozdil, Hatice Ozisik

**Affiliations:** 1 Department of Biophysics, Faculty of Medicine, Ege University, Izmir 35100, Türkiye; 2 Department of Endocrinology and Metabolism, Ege University, Izmir 35100, Türkiye; hatice.ozisik@ege.edu.tr

**Keywords:** anaplastic thyroid carcinoma, ASIC1, miR-125b-5p, miR-181b-5p, microRNA, acid-sensing ion channel

## Abstract

**Background/Objectives**: Anaplastic thyroid carcinoma (ATC) is a highly aggressive malignancy in which coordinated loss of tumor-suppressive miRNAs may contribute to reciprocal upregulation of shared target genes. We aimed to identify common miRNA targets associated with ATC and to prioritize candidates showing reproducible expression across independent datasets. **Methods**: We analyzed the matched miRNA and mRNA expression data from GSE185719, focusing on hsa-miR-125b-5p and hsa-miR-181b-5p because both were consistently reduced in ATC compared with non-malignant thyroid tissue, PTC, and FTC. Candidate targets predicted by miRWalk and supported by TargetScan and miRDB were filtered for reciprocal mRNA upregulation in ATC, then evaluated in the independent GSE33630 cohort, followed by functional enrichment and exploratory ROC analyses. **Results**: Both hsa-miR-125b-5p and hsa-miR-181b-5p were consistently reduced in ATC. Their predicted target sets overlapped at 55 genes, of which 13 were upregulated in ATC and seven remained consistently increased across all comparisons. ASIC1 was one of the strongest candidates, was independently elevated in GSE33630, and showed exploratory discrimination of ATC from normal thyroid and PTC (AUC 0.903 and 0.848, respectively). Both miRNAs were predicted to target ASIC1, although these interactions remain unvalidated. **Conclusions**: Given the proton-sensing function of ASIC1 and the importance of extracellular acidosis in aggressive tumors, these findings provide a focused hypothesis linking post-transcriptional deregulation with acid-sensing biology in ATC. Direct miRNA–ASIC1 regulation and its functional consequences require experimental validation.

## 1. Introduction

Anaplastic thyroid carcinoma (ATC) represents one of the most aggressive forms of human malignancy [1]. Although it accounts for only a small proportion of thyroid cancers, it contributes disproportionately to thyroid cancer mortality because of its extremely rapid growth, extensive local invasion, early metastatic potential, and resistance to conventional treatment. ATC is considered stage IV disease at diagnosis and often presents with a rapidly enlarging neck mass accompanied by compressive symptoms involving the trachea or esophagus. Molecularly targeted therapies, particularly combined BRAF and MEK inhibition in tumors carrying the BRAF V600E mutation, have improved outcomes for selected patients; nevertheless, ATC remains a major therapeutic challenge, and many tumors lack durable actionable responses [2]. Current molecular evidence supports a multistep model in which progressive accumulation of alterations such as TP53 and TERT promoter mutations [3], together with MAPK and PI3K-AKT pathway activation, contributes to dedifferentiation and acquisition of the anaplastic phenotype [1,4,5].

The transition from differentiated thyroid carcinoma to ATC is not explained by genomic alterations alone. ATC is also characterized by extensive transcriptional, epigenetic, metabolic, and post-transcriptional remodeling. Comparative expression studies have demonstrated marked changes in pathways related to proliferation, epithelial to mesenchymal transition, invasion, metabolism, inflammatory signaling, and loss of thyroid differentiation. The GSE33630 study, for example, showed a broad switch in mRNA expression between differentiated and anaplastic thyroid carcinomas and identified transcriptional features associated with the highly aggressive ATC phenotype [6,7]. More recently, paired small-RNA and mRNA sequencing has demonstrated that miRNA deregulation represents another major layer of ATC-associated transcriptomic reprogramming [8]. Misiak et al. performed comparative miRNA and mRNA sequencing in a cohort of 28 primary thyroid samples, including 10 ATCs, 6 PTCs, 6 FTCs, and 6 non-malignant thyroid tissues. Their analysis identified 85 miRNAs significantly deregulated in ATC, including 62 downregulated miRNAs, and demonstrated that altered miRNA abundance is associated with extensive reorganization of the ATC transcriptome. Importantly, this work established that loss of miRNA-mediated repression can contribute to increased expression of oncogenic transcripts in ATC, providing a strong rationale for examining reciprocal miRNA–mRNA expression patterns rather than evaluating miRNAs or mRNAs independently [8]. The publicly available dataset generated by that study, GSE185719, therefore provides an especially useful resource for identifying candidate regulatory relationships associated with the anaplastic state [8].

Among the miRNAs represented in this dataset, hsa-miR-125b is particularly relevant to ATC biology. Experimental studies have previously shown that hsa-miR-125b expression is reduced in ATC tissues and cell lines and that restoration of hsa-miR-125b suppresses migration and invasion. PIK3CD has been validated as a direct miR-125b target in ATC, and miR-125b restoration was associated with decreased PI3K, phosphorylated AKT, and phosphorylated mTOR signaling [9]. These observations provide direct biological support for a tumor-suppressive role of hsa-miR-125b in ATC and suggest that its loss may release additional transcripts from post-transcriptional control.

The role of miR-181b in thyroid malignancy appears more context dependent. In PTC, miR-181b has been reported to be elevated compared with adjacent normal tissue, and experimental inhibition of miR-181b reduced proliferation and promoted apoptosis through derepression of CYLD [10]. By contrast, the GSE185719 ATC cohort shows a marked reduction in hsa-miR-181b-5p not only relative to normal thyroid tissue but also relative to PTC and FTC. This difference is biologically plausible because ATC represents a highly dedifferentiated molecular state rather than simply a quantitatively more aggressive form of PTC. The concordant reduction in hsa-miR-125b-5p and hsa-miR-181b-5p therefore raises the possibility that these miRNAs may converge on a common subset of predicted target transcripts showing reciprocal upregulation in the anaplastic state.

One candidate emerging from such a regulatory relationship may be particularly relevant to the physical microenvironment of ATC. Acid-sensing ion channel 1 (ASIC1), historically annotated as ACCN2, encodes a proton-gated cation channel belonging to the degenerin/epithelial sodium channel family. ASIC1 is best characterized in the nervous system, where extracellular acidification activates channel opening, but increasing evidence indicates that ASIC1 can also be expressed in malignant cells. Tumor acidosis results from metabolic reprogramming, insufficient perfusion, hypoxia, and increased proton production and export. ASIC1 and related proton sensors have consequently been investigated as mechanisms through which malignant cells perceive and respond to an acidic extracellular environment [11].

ASIC1-associated signaling has been linked experimentally to migration, invasion, proliferation, oxidative stress, NF-κB activity, AKT signaling, and epithelial-to-mesenchymal transition in several cancer models [12,13]. At the same time, recent reports have emphasized that many of these observations remain tumor-type specific and that the downstream consequences of ASIC activation should not be generalized without experimental validation [14]. Pan-cancer analyses have reported increased ASIC1/ACCN2 expression in TCGA thyroid carcinoma compared with normal thyroid tissue, but TCGA thyroid cancer data are dominated by differentiated tumors and do not specifically resolve the ATC phenotype. Thus, whether ASIC1 is selectively associated with anaplastic transformation remains largely unexplored [15].

Based on these observations, the present study aimed to identify shared predicted targets of hsa-miR-125b-5p and hsa-miR-181b-5p that show increased expression in ATC and to independently evaluate candidate genes potentially associated with the anaplastic phenotype. To address this aim, target prediction was integrated with matched mRNA expression data from GSE185719, followed by independent evaluation in GSE33630. The analysis was designed to generate a biologically coherent and experimentally testable hypothesis rather than to establish direct miRNA–mRNA regulation.

## 2. Materials and Methods

### 2.1. Overall Study Design

The study was designed as a stepwise, public-data-based integrative analysis linking ATC-associated miRNA loss to reciprocal mRNA upregulation. The analytical workflow consisted of five consecutive stages. First, hsa-miR-125b-5p and hsa-miR-181b-5p were evaluated in GSE185719 to confirm that both showed statistically significant and directionally consistent downregulation in ATC relative to non-malignant thyroid tissue, PTC, and FTC. Second, target genes were predicted independently for each miRNA using miRWalk and were filtered for simultaneous support from TargetScan and miRDB. Third, genes common to both miRNAs were integrated with matched mRNA differential-expression results from the GSE185719 study to identify transcripts showing reciprocal upregulation in ATC. Fourth, the strongest expression-supported candidates were evaluated in the independent GSE33630 cohort. Finally, ASIC1, which showed reproducible ATC-associated elevation, was examined in greater detail with respect to predicted miRNA-binding characteristics, functional context, and exploratory ROC performance. This workflow was intentionally designed to reduce the large number of computationally predicted miRNA-mRNA relationships through successive biological and transcriptomic filters. Importantly, the analysis was not designed to infer direct causality from expression data. Reciprocal miRNA-mRNA expression was used as a prioritization criterion, and predicted interactions were considered hypotheses requiring experimental evaluation.

The primary discovery resource was Gene Expression Omnibus accession GSE185719, generated by Misiak et al. [8]. The dataset comprises 28 primary human thyroid specimens, including 10 ATCs, 6 PTCs, 6 FTCs, and 6 non-malignant thyroid tissues. Small-RNA sequencing was performed using the Illumina HiScanSQ platform (GPL15456). The original study also generated corresponding mRNA sequencing data from the same cohort, enabling direct comparison of miRNA and mRNA alterations across thyroid tumor subtypes. The GEO record is publicly available and provides the processed differential-expression file GSE185719_DE_Thyroid_miRNA_ATC_FTC_PTC_NT_Others.csv.gz; the accompanying publication provides the matched mRNA differential-expression results used in the present analysis.

The present study used the processed differential-expression results supplied by the original investigators. Raw small-RNA reads were therefore not realigned or requantified. For each candidate miRNA, log_2_ fold-change and false discovery rate (FDR) values were extracted for ATC versus non-malignant thyroid tissue, ATC versus PTC, and ATC versus FTC. Similarly, the published mRNA differential-expression table from the same study was used for downstream target-expression filtering. This approach preserved the normalization and differential-expression framework of the original matched miRNA-mRNA study while allowing a new candidate-focused analysis to be performed.

### 2.2. Rationale and Selection of hsa-miR-125b-5p and hsa-miR-181b-5p

Selection of hsa-miR-125b-5p and hsa-miR-181b-5p was based on sequential expression and target-prediction criteria. Downregulated miRNAs in GSE185719 were first evaluated across ATC versus non-malignant thyroid tissue, PTC, and FTC. Candidate miRNAs showing significant and directionally consistent reduction were then assessed using miRWalk, with additional support from TargetScan and miRDB. Following this consensus-filtering step, hsa-miR-125b-5p and hsa-miR-181b-5p were the two miRNAs retained for subsequent shared-target analysis.

hsa-miR-125b-5p was selected as the biological anchor of the analysis because it showed consistent reduction in GSE185719 and has previous experimental support in ATC. Specifically, miR-125b has been reported to be reduced in ATC tissues and cell lines, and restoration of miR-125b suppresses migration and invasion through direct regulation of PIK3CD and the PI3K/AKT/mTOR pathway [9]. hsa-miR-181b-5p was subsequently retained as the second candidate because it showed the same direction of change as hsa-miR-125b-5p across all three GSE185719 comparisons and provided a substantial high-confidence target repertoire suitable for common-target analysis. Its contrasting behavior previously reported in PTC further suggested that its reduction could reflect regulatory rewiring associated with the anaplastic state rather than a generic thyroid cancer alteration [10].

To ensure that both candidates fulfilled the required expression criterion before target analysis, their processed GSE185719 statistics were examined across ATC versus normal thyroid, PTC, and FTC. Only after confirming significant and directionally concordant downregulation in all three comparisons were the two miRNAs advanced to common-target prediction. Thus, their selection reflects the combined expression and consensus target-prediction criteria applied in this workflow and should not be interpreted as evidence that they were necessarily the two most strongly downregulated miRNAs in the complete GSE185719 dataset.

### 2.3. Consensus Prediction of miRNA Target Genes

Target-gene predictions were obtained separately for hsa-miR-125b-5p and hsa-miR-181b-5p using the miRWalk platform. miRWalk uses a random-forest-based TarPmiR framework and can evaluate potential miRNA-binding sites across the complete transcript, including the 5′ untranslated region, coding sequence (CDS), and 3′ untranslated region. The platform also integrates predictions and validation information from additional target databases [16].

To reduce algorithm-specific false-positive predictions, miRWalk derived candidates were subjected to an additional consensus filter requiring gene-level support from both TargetScan and miRDB. Support was defined as the presence of the same gene among the predicted targets reported by each database. No additional filtering based on TargetScan context scores, miRDB target scores, site number or type, or evolutionary conservation was applied. Thus, the consensus criterion reflected agreement across the three prediction resources rather than the application of a common numerical score threshold. TargetScan predicts preferential miRNA target sites largely on the basis of seed pairing and contextual determinants of targeting efficacy, whereas miRDB uses the MirTarget machine-learning framework to predict functional miRNA targets. Agreement across miRWalk, TargetScan, and miRDB was therefore used as a practical confidence filter and was not interpreted as evidence of functional targeting [17].

Duplicate transcript-level entries were collapsed to unique gene symbols before comparing the two miRNA target sets. This procedure yielded 455 unique consensus-supported targets for hsa-miR-125b-5p and 677 for hsa-miR-181b-5p. The intersection of these sets was defined as the common consensus-predicted target set and was carried forward for expression-based filtering.

### 2.4. Integration of Predicted Targets with ATC mRNA Expression

Because both candidate miRNAs were reduced in ATC, the expected direction under a simple loss-of-repression model was increased expression of their target mRNAs. The 55 genes common to hsa-miR-125b-5p and hsa-miR-181b-5p were therefore cross-referenced against mRNA differential-expression results from the matched GSE185719 cohort. This reciprocal expression pattern was considered consistent with potential miRNA-mediated derepression but was not interpreted as evidence of direct regulatory causality.

Filtering was performed in two stages. In the first stage, a common target was retained when its mRNA showed positive log_2_ fold-change in ATC relative to all other thyroid groups combined and an FDR < 0.05. This produced a broader set of ATC-associated reciprocal-expression candidates. In the second and more stringent stage, genes were required to show positive log_2_ fold-change together with FDR < 0.05 in each individual comparison: ATC versus non-malignant thyroid, ATC versus PTC, and ATC versus FTC. This second criterion was intended to prioritize genes whose elevation was reproducible across different thyroid tissue backgrounds rather than being driven by a single comparison. The sequential reduction from the initial target predictions to the final expression-supported candidates formed the basis of the target-prioritization.

### 2.5. Characterization of Predicted miRNA–ASIC1 Interactions

After ASIC1 emerged from the transcriptomic filtering procedure, the corresponding miRWalk entries were examined at transcript level. For each miRNA-ASIC1 prediction, RefSeq transcript identifier, transcript position, binding region, miRWalk binding probability, predicted binding energy, seed information, number of paired nucleotides, and longest consecutive pairing were recorded.

The presence or absence of experimental validation in miRTarBase, as reported through miRWalk, was also checked. Because neither hsa-miR-125b-5p-ASIC1 nor hsa-miR-181b-5p-ASIC1 was experimentally validated in the retrieved records, both interactions were classified throughout the manuscript as predicted interactions.

### 2.6. Independent mRNA Evaluation Using GSE33630

Independent evaluation was performed using GEO accession GSE33630, an Affymetrix Human Genome U133 Plus 2.0 Array dataset generated on platform GPL570. The GEO series contains 105 thyroid samples comprising 11 ATCs, 49 PTCs, and 45 normal thyroid tissues. The GEO metadata report processed expression values normalized using robust multi-array average methodology [18].

The processed GEO Series Matrix was used directly; raw CEL files were not re-normalized in the present analysis. Candidate genes identified from GSE185719 were matched to GPL570 probe sets using platform annotation. For the final prioritized candidate ASIC1, expression was represented by probe 37953_s_at, historically annotated to ACCN2 and currently corresponding to ASIC1.

Expression distributions were compared between ATC and normal thyroid and between ATC and PTC. The independent dataset was used principally as a directional replication cohort rather than for discovery. Candidates that reproduced the ATC-associated increase in the independent microarray cohort were prioritized, whereas genes showing inconsistent or absent replication were not advanced as primary candidates.

Among the independently reproduced candidates, ASIC1 was prioritized for focused downstream analysis because it showed consistent ATC-associated upregulation across both datasets and provided a distinct biological link to proton sensing and the acidic tumor microenvironment. ASIC1 was selected before the ROC analysis; therefore, the ROC analysis was used only to characterize the discriminatory pattern of the preselected candidate and not to rank or select genes.

### 2.7. Functional Enrichment and STRING Network Analysis

Functional context was examined using the STRING database, version 12.0, with Homo sapiens selected as the organism. STRING integrates experimentally determined and computationally predicted protein associations and provides Gene Ontology and pathway enrichment with multiple-testing correction [19]. Two input sets were evaluated. The first contained the 13 shared miRNA targets that were significantly increased in ATC relative to the combined non-ATC groups. The second contained all 55 consensus-predicted targets shared by hsa-miR-125b-5p and hsa-miR-181b-5p. No additional first-shell interactors were added to either analysis; this prevented enrichment results from being driven by proteins introduced by STRING rather than by genes identified in the present workflow. Functional categories with FDR < 0.05 were considered statistically enriched. Because the target lists were relatively small, enrichment findings were interpreted as supportive biological context and not as independent evidence of pathway activation.

### 2.8. Statistical Analysis and ROC Analysis

For the independent GSE33630 evaluation, ASIC1 expression distributions were compared using two-sided Mann-Whitney U tests because the ATC cohort was small and no assumption of normality was required. Statistical significance was defined as *p* < 0.05. Expression values are presented descriptively as means and medians where appropriate.

Exploratory receiver operating characteristic analysis was used to determine how well ASIC1 expression separated ATC from normal thyroid and ATC from PTC. Area under the ROC curve (AUC) was calculated using ASIC1 expression as a continuous predictor. Ninety-five percent confidence intervals were estimated by bootstrap resampling with 3000 iterations. The exploratory threshold maximizing the Youden index was used to derive sensitivity and specificity.

Because both threshold selection and performance estimation were performed in the same GSE33630 cohort, these ROC analyses were not considered independent clinical validation. AUC, sensitivity, and specificity are therefore described throughout the manuscript as exploratory discriminatory performance rather than diagnostic accuracy. Final graphical representations were reproduced in R using ggplot2; expression comparisons were implemented using non-parametric procedures, and ROC analyses were reproduced using the pROC package.

### 2.9. Ethics and Data Availability

All analyses were based exclusively on publicly available, de-identified transcriptomic datasets. No new biological samples were collected, no participants were contacted, and no identifiable clinical information was accessed. Consequently, the present study represents secondary analysis of existing public data and did not involve new human-subject experimentation.

**Figure 1:**
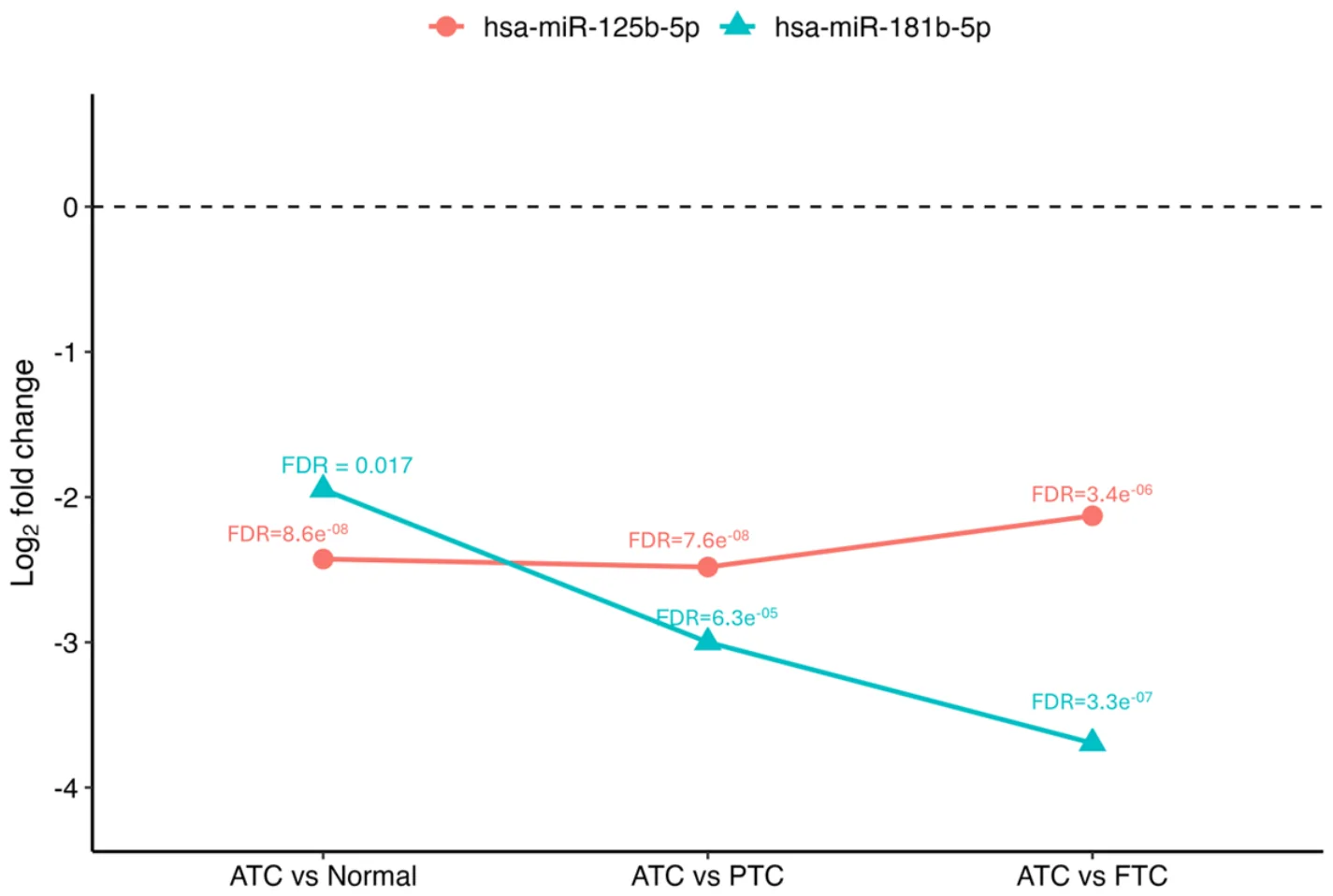
Coordinated downregulation of hsa-miR-125b-5p and hsa-miR-181b-5p in anaplastic thyroid carcinoma. The figure summarizes differential-expression results from GSE185719 and displays log_2_ fold-change values for ATC versus non-malignant thyroid tissue, PTC, and FTC. Sample-level expression distributions are not shown because the present analysis used the processed differential-expression results provided by the original study rather than reanalyzing raw sequencing data. Both miRNAs show significant and directionally consistent reductions across all three comparisons. FDR-adjusted *p* values are displayed for each comparison.

**Figure 2:**
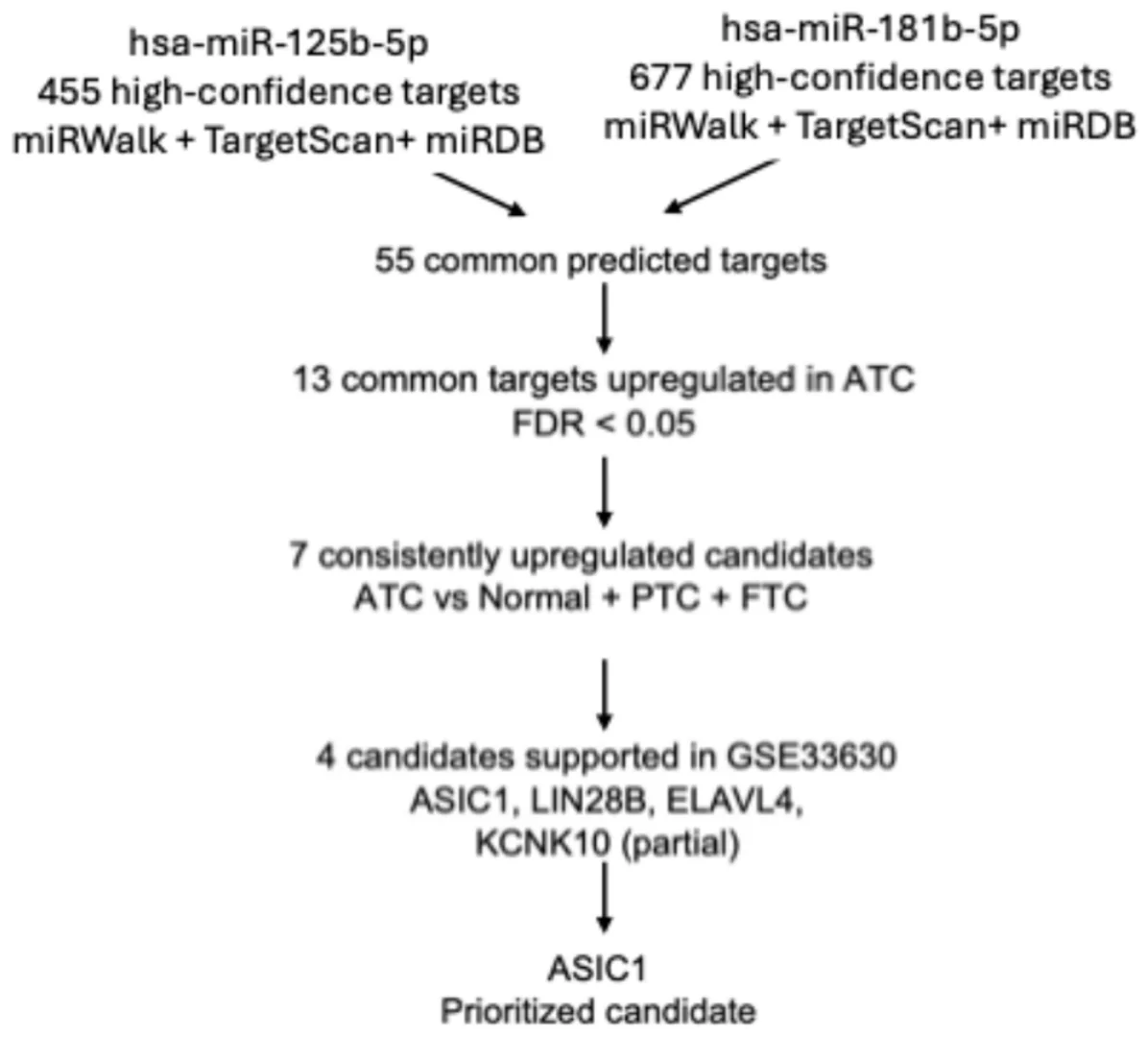
Integrative workflow used to prioritize common miRNA targets associated with ATC. Consensus target prediction identified 455 high-confidence targets for hsa-miR-125b-5p and 677 for hsa-miR-181b-5p after requiring support from miRWalk, TargetScan, and miRDB. Fifty-five genes were shared by the two miRNAs. Integration with GSE185719 mRNA expression identified 13 common targets significantly increased in ATC and seven genes consistently increased relative to normal thyroid, PTC, and FTC separately. Independent evaluation in GSE33630 prioritized ASIC1, LIN28B, and ELAVL4, with partial support for KCNK10. ASIC1 was selected as the principal candidate for further analysis.

**Figure 3:**
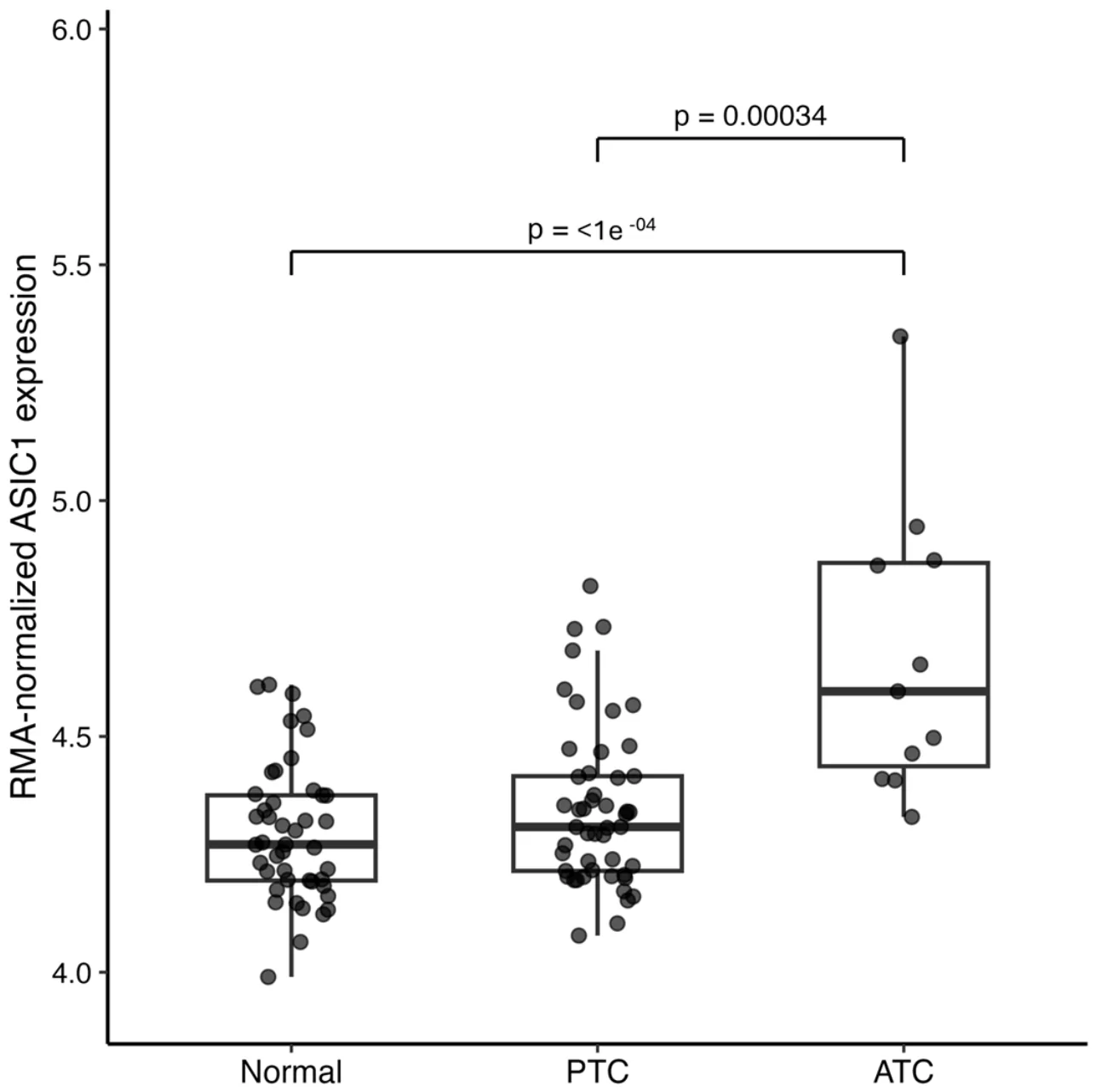
Independent replication of increased ASIC1 expression in GSE33630. Distribution of RMA-normalized ASIC1 expression measured using Affymetrix probe 37953_s_at in normal thyroid tissues (n = 45), papillary thyroid carcinomas (n = 49), and anaplastic thyroid carcinomas (n = 11). Individual observations are displayed together with group distributions. ASIC1 expression was significantly greater in ATC than in normal thyroid and PTC using two-sided Mann-Whitney U tests.

**Figure 4:**
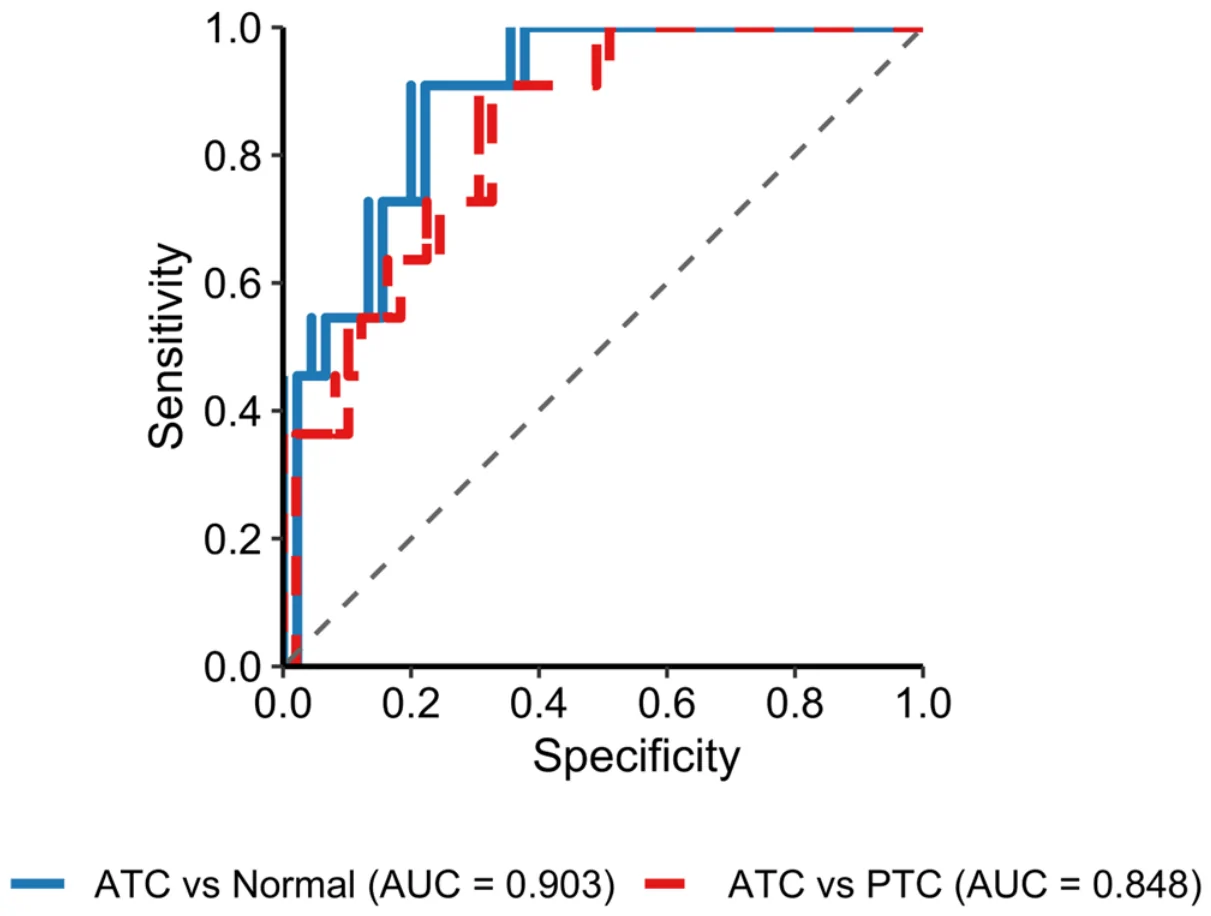
Exploratory ROC analysis of ASIC1 expression for discrimination of ATC from normal thyroid and PTC. Receiver operating characteristic curves show the ability of ASIC1 expression to distinguish ATC from normal thyroid and ATC from PTC. The AUC was 0.903 for ATC versus normal thyroid and 0.848 for ATC versus PTC. Confidence intervals were estimated using 3000 bootstrap resamples. Performance estimates are exploratory because threshold selection and evaluation were performed within the same cohort.

GSE185719 and GSE33630 are publicly available through the NCBI Gene Expression Omnibus. The source-data tables used to reproduce Figure 1, Figure 2, Figure 3 and Figure 4 via R software (version 4.4.1 GUI 1.80 Big Sur Intel build).

ChatGPT (OpenAI GPT-5.6 Sol) was used to assist with language refinement and manuscript organization. No AI tools were used to generate or alter the underlying data or to perform the reported analyses. All AI-assisted content was reviewed and verified by the authors.

## 3. Results

### 3.1. Candidate-miRNA Selection Identifies Concordant Loss of hsa-miR-125b-5p and hsa-miR-181b-5p in ATC

The first step was to verify whether the two candidate miRNAs fulfilled the directional criterion required for a common reciprocal-expression analysis. Both miRNAs showed lower expression in ATC across all three comparisons examined in GSE185719, and the magnitude of reduction was substantial.

For hsa-miR-125b-5p, expression was decreased in ATC relative to non-malignant thyroid tissue with a log_2_FC of −2.426 and an FDR of 8.60 × 10^−8^. A similar reduction was observed relative to PTC (log_2_FC = −2.482, FDR = 7.57 × 10^−8^) and FTC (log_2_FC = −2.129, FDR= 3.40 × 10^−6^). Thus, lower hsa-miR-125b-5p expression was not restricted to the comparison between cancer and normal thyroid but was also characteristic of ATC relative to two differentiated thyroid carcinoma subtypes.

hsa-miR-181b-5p showed the same overall pattern. Its expression was reduced in ATC relative to non-malignant thyroid tissue (log_2_FC = −1.947, FDR = 0.0172), PTC (log_2_FC = −2.999, FDR = 6.28 × 10^−5^), and FTC (log2FC = −3.695, FDR = 3.33 × 10^−7^). The reduction relative to PTC and FTC was particularly marked, supporting the interpretation that loss of hsa-miR-181b-5p is associated with the anaplastic transcriptional state rather than being a general feature shared by all thyroid carcinomas. Taken together, these data provided the required basis for the subsequent analysis. Two miRNAs with concordant ATC-associated loss were available for evaluation of shared targets showing reciprocal mRNA upregulation. These expression patterns are summarized in Figure 1.

### 3.2. Consensus Target Prediction Identifies 55 Genes Shared by the Two Downregulated miRNAs

The miRWalk-derived target sets were filtered to retain only genes simultaneously supported by TargetScan and miRDB. After removal of transcript-level duplicates, 455 unique genes were retained for hsa-miR-125b-5p and 677 unique genes for hsa-miR-181b-5p.

Comparison of the two sets identified 55 common predicted targets (ACSL6, ADAM11, ANKRD50, ASIC1, ASXL3, ATXN1, ATXN7, BAG4, BCL2, CBX7, CECR2, DNAL1, DUSP6, ELAVL4, ETS1, ETV6, FNDC3B, GRB10, IGSF11, INO80D, IPMK, JADE2, KCNK10, LCLAT1, LCOR, LIN28A, LIN28B, MAP3K9, NFIB, NIPAL4, NR6A1, NRXN1, PAFAH1B1, PODXL, PPP1R12B, PROX1, QKI, RFX3, RORA, SEL1L, SEMA4C, SLC16A6, SLC4A10, SOCS4, STC1, TBC1D1, TGFBR1, TRIM71, YOD1, YWHAG, ZBTB34, ZBTB7A, ZFP62, ZNF699, ZNF704). These genes represented the candidate pool most compatible with convergent regulation by the two miRNAs. Importantly, no mRNA expression criterion had yet been applied at this stage. The 55-gene intersection therefore represented a prediction-based regulatory space rather than a set of ATC-associated genes.

This distinction was important because a predicted interaction alone does not imply that the gene is transcriptionally relevant in a specific tumor type. The next stage therefore integrated the target predictions with actual ATC mRNA behavior.

### 3.3. Reciprocal miRNA–mRNA Filtering Reduces the Shared Target Set from 55 Genes to Seven Consistent ATC-Associated Candidates

When the 55 shared predicted targets were matched to GSE185719 mRNA data, 13 genes (LIN28B, ASIC1, TRIM71, ADAM11, KCNK10, ELAVL4, SLC4A10, ACSL6, LIN28A, ZBTB7A, NIPAL4, SEMA4C, and YWHAG) showed significant upregulation in ATC relative to all other thyroid groups combined. The stricter requirement that a gene also be significantly elevated in ATC relative to normal thyroid, PTC, and FTC separately reduced this set to seven candidates (LIN28B, ASIC1, TRIM71, ADAM11, KCNK10, ELAVL4, and ZBTB7A). The expression profiles of these seven candidates across the three comparisons are summarized in Table 1.

ASIC1 was notable within this set because its increase was large in all three comparisons. ASIC1 expression was strongly increased in ATC versus normal thyroid (log_2_FC = 4.55), with substantial increases also observed relative to PTC and FTC. When ATC was compared with all other thyroid groups collectively, ASIC1 remained strongly elevated (log_2_FC = 3.451, FDR = 6.10 × 10^−8^).

The sequential filtering process from 455 and 677 individual targets to 55 common targets, 13 expression-supported targets, seven consistently increased candidates, and, ultimately, the independently reproducible candidates, is summarized in Figure 2.

### 3.4. ASIC1 Is a Convergent Predicted Target of hsa-miR-125b-5p and hsa-miR-181b-5p

Transcript-level inspection of the miRWalk results provided additional support for convergence of the two miRNAs on ASIC1, although neither interaction has been experimentally demonstrated. For hsa-miR-125b-5p, one high-confidence interaction was identified in transcript NM_001256830, spanning positions 243–259 within the ASIC1 coding sequence. The miRWalk binding probability was 0.846, with a predicted binding energy of −21.7 kcal/mol. Fourteen nucleotide pairings were predicted across the interaction, including a longest consecutive pairing of 11 nucleotides. The interaction was independently flagged by both TargetScan and miRDB. For hsa-miR-181b-5p, a predicted ASIC1 interaction was identified with a miRWalk binding probability of 0.833 and predicted binding energy of −20.9 kcal/mol. The interaction contained 20 predicted pairings, including a longest consecutive matched region of 14 nucleotides. Equivalent sequence interactions were represented in the RefSeq transcripts NM_001256830, NM_020039, and NM_001095. These entries represent the same sequence-level interaction present across multiple ASIC1 transcripts rather than three biologically independent binding sites.

Notably, the predicted sites for both miRNAs were located within the coding sequence rather than the canonical 3′UTR, and neither interaction was annotated as experimentally validated in miRTarBase. These findings therefore support ASIC1 as a convergent computationally predicted target, but they do not demonstrate direct miRNA-mediated regulation.

### 3.5. Independent GSE33630 Analysis Reproduces ASIC1 Upregulation in ATC

The seven stringent candidates were next examined in the independent GSE33630 cohort. Cross-dataset evaluation showed reproducible elevation of ASIC1, LIN28B, and ELAVL4, whereas KCNK10 showed partial replication and the remaining candidates were less consistent across datasets. Among these reproduced candidates, ASIC1 was taken forward for focused analysis based on the combination of cross-dataset expression consistency and its biological relevance to proton-sensing mechanisms.

In GSE33630, mean RMA-normalized ASIC1 expression was 4.671 in ATC compared with 4.296 in normal thyroid and 4.347 in PTC. The difference between ATC and normal thyroid was highly significant using the two-sided Mann–Whitney U test (*p* = 4.06 × 10^−5^). ASIC1 expression was also significantly greater in ATC than in PTC (*p* = 3.54 × 10^−4^). The independent evaluation is particularly relevant because the two datasets were generated using different transcriptomic technologies. GSE185719 was based on sequencing, whereas GSE33630 used the Affymetrix GPL570 microarray platform. Reproduction of the same directional ASIC1 increase across these independent cohorts and platforms reduced the likelihood that the observation resulted from a single dataset-specific technical effect. The distribution of ASIC1 expression across the three tissue groups is shown in Figure 3.

### 3.6. Exploratory ROC Analysis of ASIC1 Expression in ATC

Because ASIC1 was reproducibly increased in ATC, we next examined whether its expression carried information capable of separating the anaplastic samples from other thyroid tissues in the independent GSE33630 evaluation cohort. For ATC versus normal thyroid, ASIC1 produced an AUC of 0.903 with a bootstrap 95% confidence interval of 0.801–0.975. The Youden-derived exploratory expression threshold was approximately 4.407, corresponding to a sensitivity of 90.9% and specificity of 80.0%. For ATC versus PTC, ASIC1 produced an AUC of 0.848 with a 95% confidence interval of 0.714–0.947. At the corresponding exploratory threshold, sensitivity was 90.9% and specificity was 69.4%. Thus, ASIC1 showed stronger separation of ATC from normal thyroid than from PTC but retained substantial discriminatory information in both comparisons. These findings support its association with the anaplastic phenotype. However, because the thresholds were selected and evaluated using the same GSE33630 samples, the values represent exploratory performance rather than externally validated diagnostic sensitivity and specificity. Because the cut-offs were optimized in the same cohort, these sensitivity and specificity estimates are likely optimistic and should not be interpreted as expected performance in an independent population. The corresponding ROC curves are shown in Figure 4.

### 3.7. Functional Enrichment Supports miRNA-Regulatory Reprogramming but Does Not Identify a Single Dominant Cancer Pathway

STRING analysis of the 13 ATC-upregulated common targets identified several statistically enriched biological processes related to miRNA regulation. Significant terms included miRNA metabolic process (FDR = 0.0054), miRNA processing (FDR = 0.0135), regulation of miRNA-mediated gene silencing (FDR = 0.0135), and miRNA catabolic process (FDR = 0.0314). The enriched biological processes and corresponding FDR values are summarized in Table 2.

Inspection of the associated STRING network indicated that this signal was driven largely by the LIN28A/LIN28B/TRIM71 regulatory module. The enrichment of miRNA-processing and miRNA-metabolic terms also raises the possibility of a regulatory feedback structure in which miRNAs target components that themselves influence miRNA biogenesis or activity. However, the present enrichment analysis cannot establish the existence or direction of such a feedback loop, and this interpretation remains hypothesis generating. Analysis of the broader 55-gene common target set produced a more heterogeneous functional profile, including enrichment of developmental processes such as central nervous system neuron differentiation and tube formation together with miRNA-associated processes. A single canonical oncogenic pathway did not dominate the analysis.

**Figure 5:**
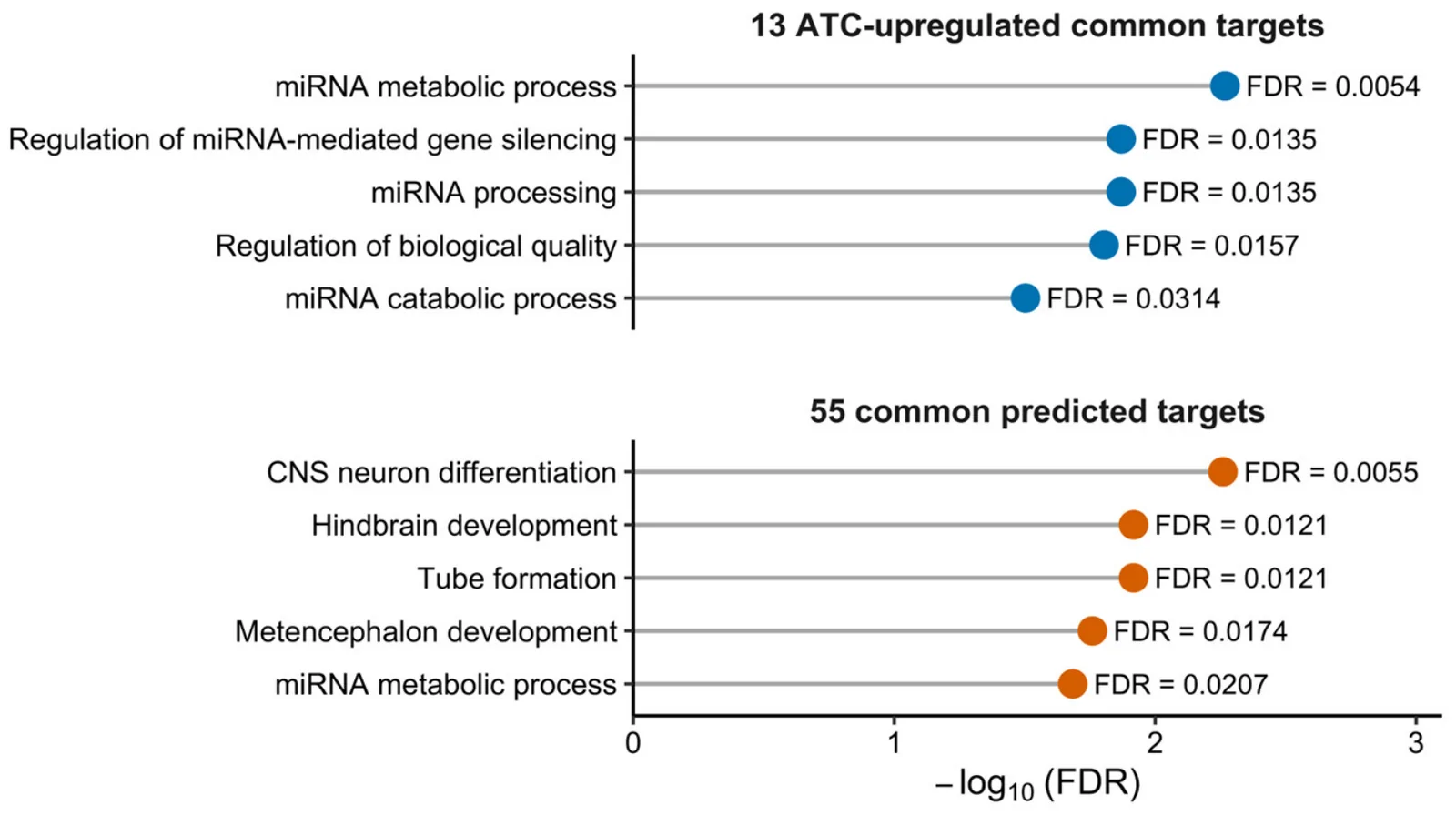
Functional enrichment analysis of shared hsa-miR-125b-5p/hsa-miR-181b-5p targets. STRING-based Gene Ontology Biological Process enrichment analysis was performed for the 13 common predicted targets significantly upregulated in ATC and for the broader set of 55 common consensus-predicted targets. The 13-gene subset was predominantly enriched for processes related to miRNA metabolism, processing, and regulation of miRNA-mediated gene silencing, whereas the 55-gene set showed a more heterogeneous profile including neuronal differentiation and developmental processes. Dot position represents −log10(FDR), with FDR-adjusted values shown for each term.

We therefore interpreted the enrichment findings conservatively. Rather than demonstrating activation of a specific signaling pathway, the results suggest that coordinated loss of the two miRNAs may affect a heterogeneous regulatory network that includes components involved in post-transcriptional control and cell-state regulation. These findings indicate that the convergent target space is functionally heterogeneous, with the strongest enrichment in the expression-supported subset involving miRNA-related regulatory processes rather than a single dominant oncogenic pathway (Figure 5).

## 4. Discussion

The present study used a focused integrative strategy to identify predicted miRNA targets showing reciprocal mRNA upregulation in association with coordinated miRNA loss in ATC. Starting from hsa-miR-125b-5p and hsa-miR-181b-5p, both of which were consistently reduced in ATC relative to normal thyroid, PTC, and FTC, we progressively narrowed several hundred predicted targets to 55 shared genes, 13 ATC-upregulated common targets, and seven candidates that remained significantly increased across each individual thyroid comparison. Independent analysis of GSE33630 subsequently prioritized ASIC1 because its ATC-associated increase was reproduced across two different cohorts and transcriptomic platforms. The central finding is therefore not simply that ASIC1 is expressed in thyroid cancer, but that ASIC1 emerged from a directional miRNA-target filtering strategy and was independently enriched in ATC compared with both non-malignant thyroid and PTC.

The behavior of miR-125b-5p provides strong biological support for the analytical framework. miR-125b has previously been experimentally characterized as a tumor-suppressive miRNA in ATC. Restoration of miR-125b decreases ATC cell migration and invasion, while PIK3CD was demonstrated by luciferase analysis to be a direct target. miR-125b restoration also attenuated PI3K, phosphorylated AKT, and phosphorylated mTOR expression [9]. Our results reproduce the marked decrease in hsa-miR-125b-5p in an independent transcriptomic context and extend the concept from a single validated target toward a broader predicted target network. Importantly, our data do not suggest that ASIC1 replaces PIK3CD as an established hsa-miR-125b target in ATC. Rather, ASIC1 represents an additional candidate whose regulatory relationship remains to be tested experimentally.

The interpretation of miR-181b-5p is more nuanced. Previous work in PTC reported increased miR-181b expression relative to adjacent normal thyroid tissue, with functional evidence that miR-181b can promote cell growth at least partly through repression of CYLD [10]. In GSE185719, however, hsa-miR-181b-5p was substantially lower in ATC than in PTC, FTC, and normal thyroid. This apparent difference should not necessarily be viewed as contradictory. ATC is characterized by major loss of differentiated thyroid identity and extensive regulatory rewiring, and miRNA behavior can shift with cellular context, tumor stage, and differentiation state. Thus, miR-181b may display oncogenic behavior in differentiated PTC while becoming part of a distinct regulatory network during progression toward the anaplastic phenotype [10].

A major strength of the present approach is that target prediction was not used in isolation. Computational miRNA prediction algorithms generate large numbers of potential interactions, many of which may not be biologically active in a particular tissue. We therefore required concordance between miRWalk, TargetScan, and miRDB and subsequently asked whether the predicted target actually behaved in the direction expected in ATC. This reduced the initial prediction space from hundreds of targets to a relatively small set of genes showing reciprocal expression. Nevertheless, consensus among prediction algorithms does not constitute experimental validation. TargetScan, miRDB, and miRWalk use different but partly overlapping sequence and statistical principles, and agreement among them increases confidence but cannot establish physical miRNA–mRNA interaction [16,17,20].

ASIC1 was particularly interesting because its biological function provides a plausible connection to a well-established property of solid tumors: extracellular acidosis [21,22]. ASIC1 is a proton-gated cation channel that responds to reductions in extracellular pH. Solid tumors commonly develop acidic extracellular environments as a consequence of increased glycolytic metabolism, hypoxia, inadequate perfusion, carbon dioxide production, and proton extrusion. Acidosis is not merely a metabolic by-product; it can impose selective pressure on malignant cells and contribute to invasion, extracellular-matrix remodeling, immune evasion, and treatment resistance [23]. Proton-sensitive channels and receptors are therefore increasingly recognized as potential interfaces between the physical tumor microenvironment and intracellular signaling [24].

Evidence from other malignancies provides a biological rationale for investigating ASIC1 in this setting. ASIC1 has been implicated in breast, hepatocellular, pancreatic, brain, and other cancer models. In breast cancer cells, for example, ASIC1 has been linked to acidosis-induced calcium signaling, reactive oxygen species generation, NF-κB activity, and tumor growth. Other studies have associated ASIC1-containing channels with migration, invasion, epithelial-to-mesenchymal transition, and intracellular pathways involving AKT or RhoA [11,15,25]. These observations support the possibility that ASIC1 could influence how malignant cells respond to acid stress. At the same time, a recent mechanistic review emphasized that some proposed ASIC-mediated cancer signaling models remain incompletely resolved, particularly because ASIC currents are often transient and their downstream calcium effects may be indirect. Consequently, the present study should be considered a rationale for ATC-specific functional experiments rather than evidence that mechanisms established in other cancers automatically apply to ATC [25].

ASIC1 is not entirely absent from previous thyroid cancer resources. Pan-cancer analyses summarized in the recent literature have reported increased ASIC1/ACCN2 expression in TCGA thyroid carcinoma relative to non-tumor tissue [15]. However, TCGA-THCA consists overwhelmingly of differentiated thyroid carcinomas and does not provide a dedicated ATC cohort. The finding therefore establishes that ASIC1 expression can occur in thyroid malignancy but does not determine whether it is preferentially associated with anaplastic transformation. The present comparison adds a different level of information: in GSE33630, ASIC1 was significantly higher in ATC than in both normal thyroid and PTC, and the same direction was observed in the independent GSE185719 cohort. This cross-histology and cross-platform consistency is one of the strongest observations supporting further investigation of ASIC1 in ATC.

The original GSE33630 study is also biologically compatible with this interpretation. Hébrant et al. described ATC as a major transcriptional program switch characterized by enhanced glycolysis, lactate production, proliferation, inflammatory signaling, EMT, invasion, and dedifferentiation [7]. Those features are compatible with a tumor environment in which pH sensing may become increasingly relevant. Nevertheless, our analysis did not directly measure extracellular pH, glycolytic flux, ion-channel activity, or acid tolerance. It would therefore be premature to conclude that ASIC1 upregulation causes adaptation to acidosis in ATC. The appropriate interpretation is that ASIC1 provides a plausible molecular link between the ATC transcriptomic phenotype and proton-sensing biology that deserves direct experimental testing.

The other expression-supported candidates also provide useful context. LIN28B was strongly increased in the discovery cohort and reproduced in the independent dataset. LIN28 proteins are well-known regulators of cellular differentiation and miRNA processing [26], particularly through control of the let-7 family, making the appearance of LIN28A/LIN28B [27] in the STRING enrichment analysis biologically coherent with the extensive post-transcriptional reprogramming observed in ATC. Its recurrence across datasets may therefore be compatible with the dedifferentiated regulatory state of ATC. TRIM71 also contributed to the miRNA-processing enrichment signal in the discovery network, although it did not reproduce sufficiently in GSE33630 to justify prioritization as the main candidate. ELAVL4 also reproduced in the independent dataset. As an RNA-binding protein involved in post-transcriptional regulation, its altered expression may represent an additional component of the broader RNA-regulatory reprogramming associated with ATC, although its specific functional role in this tumor type remains to be established. KCNK10, another ion-channel-related gene, showed a clear increase relative to normal thyroid but weaker discrimination from PTC. These observations suggest that the regulatory consequences associated with coordinated loss of the two miRNAs are unlikely to be restricted to a single gene, even though ASIC1 provided the clearest combination of expression consistency and biological interpretability.

The exploratory ROC analysis further illustrates the differential expression of ASIC1 across the examined thyroid tissue groups. Within GSE33630, ASIC1 expression yielded an AUC of 0.903 for ATC versus normal thyroid and 0.848 for ATC versus PTC, indicating substantial separation within this exploratory dataset. The lower specificity in the ATC-versus-PTC analysis is unsurprising given that ASIC1 expression is not completely absent from differentiated thyroid carcinoma and has been detected in broader thyroid cancer datasets. The ROC findings should therefore not be interpreted as evidence that ASIC1 is ready for diagnostic use. The number of ATC cases was only 11, and the threshold was optimized within the same cohort in which performance was measured. Independent validation with a larger ATC cohort would be required before clinical sensitivity, specificity, or a diagnostic cut-off could be proposed.

The predicted miRNA-binding sites themselves also warrant caution. Both miR-125b-5p and miR-181b-5p were predicted to interact with ASIC1 within the coding sequence rather than the canonical 3′UTR. miRNA interactions outside the 3′UTR, including CDS binding, have been described and are explicitly incorporated into miRWalk’s whole-transcript prediction framework [16]. Although functional miRNA interactions within coding regions have been reported, targeting of the 3′UTR remains the best-characterized mode of canonical miRNA-mediated repression. CDS-targeting interactions are comparatively less well characterized and may be more context dependent. Accordingly, the predicted CDS interactions identified here should be interpreted cautiously and require direct experimental confirmation, ideally using reporter constructs containing the relevant ASIC1 CDS regions together with sequence-mutated controls [28]. However, CDS predictions generally require stronger experimental support before functional interpretation. Neither of the ASIC1 interactions identified here was annotated as experimentally validated in miRTarBase. The present expression relationship should therefore be described as consistent with potential derepression, not as proof that decreased miR-125b-5p or miR-181b-5p directly causes ASIC1 upregulation.

Several limitations should consequently be recognized. First, the study is entirely based on previously generated transcriptomic data and contains no new wet-laboratory validation. Second, the miRNA and initial mRNA discovery analyses originate from the same GSE185719 cohort; although this is useful for identifying reciprocal expression relationships, it does not provide independent evidence of miRNA–mRNA regulation. Third, ATC is rare, and both discovery and independent evaluation cohorts contain relatively small numbers of ATC samples. Fourth, GSE185719 and GSE33630 were generated using different technologies, which is a strength in terms of cross-platform replication but also prevents direct comparison of absolute expression values. Fifth, transcript abundance does not establish ASIC1 protein abundance, membrane localization, proton-gated current, or functional channel activity. Finally, the predicted miRNA–ASIC1 relationships remain unvalidated and may represent indirect associations within a broader regulatory network.

These limitations define clear experimental next steps. ATC cell models should first be characterized for basal hsa-miR-125b-5p, hsa-miR-181b-5p, ASIC1 mRNA, and ASIC1 protein abundance. miRNA mimic experiments would then determine whether restoration of either miRNA reduces endogenous ASIC1 expression. Reporter constructs containing the predicted ASIC1 CDS interaction regions, together with sequence-mutated controls, would be required to establish direct binding. If regulation is confirmed, functional experiments performed at physiological and acidic extracellular pH could test whether ASIC1 contributes to viability, migration, invasion, calcium signaling, or stress adaptation. Pharmacological inhibition or genetic silencing of ASIC1 would provide complementary evidence for causality.

Overall, the present findings suggest that miRNA loss and acid-sensing biology may intersect in the molecular reprogramming of ATC. The study does not yet establish a functional hsa-miR-125b-5p/hsa-miR-181b-5p-ASIC1 axis, but it narrows a large computational target space to a reproducible and experimentally tractable candidate. This distinction is important: the principal contribution of the study is the generation of a focused mechanistic hypothesis supported by reciprocal expression, consensus target prediction, and independent mRNA replication.

## 5. Conclusions

This integrative analysis identifies ASIC1 as a reproducibly upregulated transcript associated with coordinated reduction in hsa-miR-125b-5p and hsa-miR-181b-5p in anaplastic thyroid carcinoma. By combining candidate-miRNA selection, consensus target prediction, reciprocal mRNA filtering, and independent cohort evaluation, the analysis reduced hundreds of predicted interactions to a small number of ATC-associated candidates. ASIC1 was distinguished by its consistent increase across independent datasets and its differential expression between ATC, normal thyroid, and PTC.

The predicted miRNA–ASIC1 interactions remain computational and should not be interpreted as evidence of direct regulation. Nevertheless, the findings provide a testable framework in which the observed reciprocal expression pattern is consistent with the possibility that reduced miRNA-mediated control may contribute to increased ASIC1 expression in the ATC phenotype, potentially linking post-transcriptional deregulation with the acidic tumor microenvironment.

## Figures and Tables

**Table 1:** Expression profiles and independent replication status of the seven stringent common-target candidates in GSE185719.

Gene	ATC vs. Normal log_2_FC	FDR	ATC vs. FTC log_2_FC	FDR	ATC vs. PTC log_2_FC	FDR	GSE33630 Replication
LIN28B	3.82	0.0119	3.76	0.0127	7.05	0.000641	Yes
ASIC1	4.55	0.000040	2.57	0.0154	3.79	0.000214	Yes
TRIM71	3.15	0.00455	4.36	0.00153	3.26	0.00570	Not consistently reproduced
ADAM11	2.28	0.00252	3.81	0.000009	3.19	0.000068	Not consistently reproduced
KCNK10	3.40	0.00507	2.49	0.0237	2.58	0.0448	Partial
ELAVL4	2.28	0.0228	2.25	0.0202	2.26	0.0260	Yes
ZBTB7A	1.40	0.00181	1.41	0.00443	1.64	0.000320	Not consistently reproduced

**Table 2:** Functional enrichment of shared hsa-miR-125b-5p/hsa-miR-181b-5p target sets.

Gene Set	Database/Category	Term	FDR
13 ATC-upregulated targets	GO Biological Process	miRNA metabolic process	0.0054
13 ATC-upregulated targets	GO Biological Process	miRNA processing	0.0135
13 ATC-upregulated targets	GO Biological Process	Regulation of miRNA-mediated gene silencing	0.0135
13 ATC-upregulated targets	GO Biological Process	Regulation of biological quality	0.0157
13 ATC-upregulated targets	GO Biological Process	miRNA catabolic process	0.0314
55 common targets	GO Biological Process	CNS neuron differentiation	0.0055
55 common targets	GO Biological Process	Tube formation	0.0121
55 common targets	GO Biological Process	Hindbrain development	0.0121
55 common targets	GO Biological Process	Metencephalon development	0.0174
55 common targets	GO Biological Process	miRNA metabolic process	0.0207

## Data Availability

The datasets analyzed in this study are publicly available in the NCBI Gene Expression Omnibus under accession numbers GSE185719 and GSE33630. The processed data and analysis files generated in this study are available from the corresponding author upon reasonable request.

## Abbreviations

The following abbreviations are used in this manuscript:

ACCN2	Amiloride-sensitive cation channel 2
ASIC1	Acid-sensing ion channel 1
ATC	Anaplastic thyroid carcinoma
AUC	Area under the receiver operating characteristic curve
CDS	Coding sequence
EMT	Epithelial-to-mesenchymal transition
FDR	False discovery rate
FTC	Follicular thyroid carcinoma
GEO	Gene Expression Omnibus
GO	Gene Ontology
miRNA	MicroRNA
mRNA	Messenger RNA
NCBI	National Center for Biotechnology Information
PTC	Papillary thyroid carcinoma
RMA	Robust multi-array average
ROC	Receiver operating characteristic
TCGA	The Cancer Genome Atlas
UTR	Untranslated region
